# Phenolic Plant Extracts Induce Sirt1 Activity and Increase Antioxidant Levels in the Rabbit's Heart and Liver

**DOI:** 10.1155/2018/2731289

**Published:** 2018-07-04

**Authors:** G. Corbi, V. Conti, K. Komici, V. Manzo, A. Filippelli, M. Palazzo, F. Vizzari, S. Davinelli, A. Di Costanzo, G. Scapagnini, N. Ferrara, D. Casamassima

**Affiliations:** ^1^Department of Medicine and Health Sciences, University of Molise, Campobasso, Italy; ^2^Department of Medicine and Surgery, University of Salerno, Salerno, Italy; ^3^Department of Agricultural, Environmental and Food Sciences, University of Molise, Campobasso, Italy; ^4^Department Translational Medical Sciences, University of Naples Federico II, Naples, Italy

## Abstract

**Background:**

Several dietary phytochemicals potentially regulate the equilibrium between oxidant and antioxidant species. The aim of this study was to evaluate the effects of *Lippia citriodora*, *Raphanus sativus*, and *Solanum lycopersicum* on blood parameters, oxidative/antioxidant status, and SIRT1 activity in the rabbit's heart and liver.

**Methods:**

Twenty rabbits were divided into 4 groups of 5 animals each. The control group (CN) received a feed without any additives. One intervention group received a supplement containing verbascoside (VB), another *Raphanus sativus* extract (RAP), and lastly lycopene (LYC). Oxidant-antioxidant parameters and SIRT1 activity were measured in plasma and in the heart and liver, respectively.

**Results:**

The treatment with VB, RAP, and LYC resulted in a marked improvement in the blood lipid and glycaemic profile in respect to CN. VB was the most effective, but all three plant extracts induced a significant reduction in oxidant parameters as well as an increase in antioxidant tissue activity and vitamin A and E levels. SIRT1 activity was significantly increased in both VB and LYC compared to CN, but the increased levels in the VB group were far the highest. The multivariate analysis suggests that the benefits of VB, particularly the antiglycaemic and antioxidant effects, might be mediated by increasing SIRT1 activity.

## 1. Introduction

Aging is a phenomenon universally involving all organisms, and it is characterized by a progressive decline of physiological function, mainly the cardiovascular and metabolic profile, leading to death. In the last decades, the prevalence of the aging population is rising progressively, and this “aging boom” has led to augmented investigation in the mechanisms involved in the aging genesis and progression [1]. A global consensus identifies aging as a multifactorial process genetically determined and epigenetically influenced by the environment. However, many theories of aging have been proposed, most of them considering the reactive oxygen species (ROS) and the oxidant/antioxidant equilibrium as crucial protagonists in aging genesis and progression [2]. Several studies have suggested that the increased ROS induce cellular damage and mitochondrial dysfunction influences the aging process. Based on these results, interesting models focused on antioxidant supplementation as an antiaging strategy have been developed [3–5].

Sirtuins, nicotinamide adenine dinucleotide- (NAD-) dependent protein deacetylases, have shown to mediate many pathways in metabolism, cardiovascular disease, and longevity of mammalian organisms [6]. In a broad range of experimental models, sirtuins proved to play a very important role against oxidative damage [7]. For instance, some studies have suggested that sirtuins deacetylate the antioxidant enzyme manganese superoxide dismutase (SOD2) in the mitochondrial matrix, resulting in increased scavenging of ROS [8]. Other interesting studies have described that the expression of several antioxidant genes such as superoxide dismutase, catalase, peroxiredoxins 3 and 5, thioredoxin 2, and thioredoxin reductase 2 is regulated by sirtuin isoform 1 (SIRT1) through deacetylation of the transcription factor FoxO3a and the transcriptional coactivator peroxisome proliferator-activated receptor *γ*-coactivator 1*α* (PGC-1α) pathway [9, 10]. The emerging data on the beneficial effects of sirtuin activators on aging and age-related diseases have focused on sirtuins as a new potential target [11, 12]. In fact, SIRT1 activators are intensively investigated for their ability to treat metabolic disorders, inflammation, and endothelial dysfunction, with a possible implication in the clinical therapy management [13, 14]. Recently, the use of dietary plant phenolic extracts is becoming an attractive alternative therapy. In fact, the use of these compounds has been mainly recognized as part of dyslipidaemia treatment and vascular wall health [15], as well as of neuroprotection [16]. The most interesting plant extract components that could have a therapeutic application are lycopene (*Solanum lycopersicum* L.), horseradish (*Raphanus sativus* L.), and verbascoside (*Lippia citriodora*) [17]. It has been previously demonstrated that a dietary supplementation with lycopene, horseradish, and verbascoside has a positive effect on plasma oxidative status in an animal model of adult rabbits. In addition, Campo and colleagues [18, 19] have reported that the administration of verbascoside in subjects with cardiovascular risk factors reduced the platelet aggregation values. Although the modulation of the biochemical profile and oxidative status seems to be strongly associated with sirtuin activity and phenolic extracts have some beneficial effects on it, surprisingly, no data are reported on the role of phenolic components in sirtuin activity. In the present study, we aimed to evaluate the dietary effects of plant extracts, *Lippia citriodora* (verbascoside), horseradish (*Raphanus sativus L*.), and lycopene (*Solanum lycopersicum L*.), on blood parameters, plasma oxidant/antioxidant status, and SIRT1 activity in the rabbit's heart and liver tissues.

## 2. Methods

### 2.1. Animals and Experimental Design

All breeding procedures and the management of animals were conducted in compliance with the European directive 2010/63/EU, concerning the protection of animals used for scientific purposes. The Ethic and Scientific Committee of the University of Molise approved the full experimental design. The experiment was conducted on 20 weaned male rabbits (New Zealand white and Californian) and lasted 80 days. The animals were reared in cages (two per cage) equipped with feeders and automatic watering. Temperature and relative humidity of the rabbitry were recorded continuously using a digital thermograph located at the height of the cages. The rabbitry was equipped with an environmental microclimate control system in order to maintain a temperature of 18 ± 4°C and a relative humidity of 70 ± 5% throughout the experimental period.

Rabbits were randomly divided into four groups of 5 animals each, matched by age (38 ± 2 days) and body weight (1.49 ± 0.07 kg). The control group (CN) received a fattening feed without any natural supplements, conversely to the other experimental groups. One intervention group received a supplement of *Lippia citriodora* extract, containing verbascoside 5 mg/kg feed (VB group), another group received 350 mg of root extract of *Raphanus sativus*/kg feed (RAP group), and the last one received 5 mg of lycopene, extracted from tomato fruit/kg feed (LYC group). The feed was administered ad libitum and was produced by Agrizoo s.n.c. (Miranda, Isernia, Italy). Feed additives based on *Lippia citriodora* (0.5% of verbascoside as the main component), horseradish (*Raphanus sativus* dry root extract with enzyme complex Inuzyme®), and lycopene (2% of *Solanum lycopersicum* extract fruit) were provided by Sintal Zootecnica (Isola Vicentina, Vicenza, Italy), Erba Vita Italia S.p.A. (Montegrimano Terme, Perugia, Italy), and Erbamea srl (San Giustino, Perugia, Italy), respectively.

### 2.2. Sampling and Laboratory Analysis of Blood

Individual samples were taken from the auricularis marginalis vein with the vacutainer method (Venoject, Terumo Europe N.V., Leuven, Belgium) using tubes with lithium heparin for plasma production. Blood samplings were performed at the beginning (weaning age, 0 d) and at the half (40 d) and at the end of the experiment (80 d). Blood was centrifuged for 20 min at 3000 rpm, and the following parameters were determined on the plasma: glucose, triglycerides, total cholesterol, low-density lipoprotein (LDL) cholesterol, high-density lipoprotein (HDL) cholesterol, serum glutamic oxaloacetic transaminase (sGOT), serum glutamic pyruvic transaminase (sGPT), bilirubin, and creatinin, using a semiautomatic clinical chemistry analyzer Arco model (Biotechnical Instruments, S.p.A., Italy). Reactive oxygen metabolites (ROMs) were spectrophotometrically determined with the colorimetric method proposed by Diacron at a wavelength of 505 nm using a specific commercial kit [20]. The results were expressed in Carr units (1 Carr corresponds to 0.024 mmol/l of H_2_O_2_).

The determination of thiobarbituric acid reactive substances (TBARS) was spectrophotometrically performed according to Esterbauer and Zollern [21] using a standard curve with 1,1,3,3-tetramethoxypropane (Sigma-Aldrich, St. Louis, USA). The results were expressed as *μ*mol of malondialdehyde (MDA)/l of plasma. Vitamins A and E were extracted from plasma samples with chloroform, according to Zhao et al. [22]. Vitamin amount was detected by HPLC (Kontron Instruments, Italy) which consisted of an automatic autosampler (HPLC Autosampler 360) with a loop of 20 *μ*l, pump system (HPLC Pump 422), and a column C18 (5 *μ*m, 250 × 4.60 mm) (Phenomenex, Torrance, CA, USA). The mobile phase consisted of a mixture of acetonitrile and methanol (85 : 15 *v*/*v*) with a flow value of 1 ml/min. Vitamins A and E were identified by comparing the retention time of the samples with the retention time of the pure standards (>97%) purchased by Sigma-Aldrich (St. Louis, USA). The quantification was performed using the Gyminix system (version 1.8.1) by comparing the peak of the area with that of the reference standard curve.

### 2.3. Tissue Processing and Homogenate Preparation

At the end of the study period, all the rabbits were sacrificed, and their hearts and liver tissue were immediately removed and rinsed free of blood. The left ventricle was separated, frozen in liquid nitrogen, and stored at −80°C until processing. The tissues were homogenized in lysis buffer containing 10 mM Tris-HCl (pH 7.4); 0.5% NP-40; 250 mM sucrose; 0.1 mM EGTA; 10 mM NaCl; 15 mM MgCl2; 1 mM PMSF; 1 *μ*g/ml each of aprotinin, leupeptin, and pepstatin; 1 mM Na3VO4; and 1 mM NaF.

After 1 h, the homogenate was obtained by centrifugation at 14000 rpm for 10 min at 4°C. Protein concentration was determined using the Bio-Rad assay (Milan, Italy) [23].

### 2.4. Total Antioxidant Activity (TAC)

The TAC of heart and liver supernatants was measured using the ability of endogenous antioxidants to scavenge the 2,2′-azinobis(3-ethylbenzothiazoline-6-sulfonic acid) (ABTS) radical cation decolorization assay, according to the method of Re et al. [24]. The ABTS radical was generated by chemical reaction with potassium persulphate. For this purpose, 25 ml of ABTS (7 mM) was spiked with 440 *μ*l of potassium persulphate (140 mM) and allowed to stand in darkness at room temperature for 12–16 h (time required for the formation of the radical). Trolox was used as the standard, and the total antioxidant capacity of samples was defined as the concentration of Trolox having equivalent activity which is expressed as *μ*mol/g tissue weight. Each experiment was performed in triplicate.

### 2.5. Isolation and Extraction of Nuclei for SIRT1 Deacetylase Assay

Aliquots of heart and liver tissue homogenate (without protease inhibitors) were spun through 4 ml of 30% sucrose, 10 mM Tris-HCl (pH 7.5), 10 mM NaCl, and 3 mM MgCl2 at 1300 ×g for 10 min at 4°C; the pellet was washed with cold 10 mM Tris-HCl (pH 7.5) and 10 mM NaCl. The nuclei were suspended in 50–100 *μ*l of extraction buffer containing 50 mM Hepes KOH (pH 7.5), 420 mM NaCl, 0.5 mM EDTA Na_2_, 0.1 mM EGTA, and 10% glycerol, sonicated for 30 s, and stood on ice for 30 min.

After centrifugation at 13000 rpm for 10 min, an aliquot of the supernatant (crude nuclear extract) was used to determine protein concentration using the Bio-Rad assay [23].

### 2.6. SIRT1 Deacetylase Expression and Activity

SIRT1 protein expression was measured using the Human SIRT1 (sirtuin 1) ELISA Kit (Elabscience® Biotechnology Inc., United States). Briefly, serially diluted standards or samples were added to the micro ELISA plate wells, precoated with an antibody specific to human SIRT1. Then, a biotinylated detection antibody specific for human SIRT1 and avidin-horseradish peroxidase (HRP) conjugate were added to each microplate well and incubated. After several washing steps, the substrate solution was added to each well. Lastly, the enzyme-substrate reaction was terminated by the addition of stop solution producing a colored reaction that was measured spectrophotometrically at a wavelength of 450 ± 2 nm. The optical density (OD) values were proportional to the concentration of human SIRT1, and the concentration of human SIRT1 in the samples was calculated by comparing the OD of the samples to the standard curve.

The SIRT1 deacetylase activity was evaluated in the crude nuclear extract from heart and liver tissues of all the rabbit groups. We measured SIRT1 using a deacetylase fluorometric assay kit (Sir2 Assay Kit, CycLex, Ina, Nagano, Japan). The final reaction mixture (100 *μ*l) contained 50 mM Tris-HCl (pH 8.8), 4 mM MgCl2, 0.5 mM DTT, 0.25 mA/ml lysyl endopeptidase, 1 *μ*M Trichostatin A, 200 *μ*M NAD, and 5 *μ*l of nuclear samples.

The fluorescence intensity at 440 nm (exc. 340 nm) was measured every 30 s for a total of 60 min immediately after the addition of fluorosubstrate peptide (20 *μ*M final concentration) and normalized by protein concentration. All determinations were performed in triplicate on 10 different samples, and the results are reported as relative fluorescence/*μ*g of protein (AU) [23]. Each experiment was performed in triplicate.

### 2.7. Statistical Analysis

Blood parameters were assessed using the delta (difference between post- and pretreatment levels). After the evaluation of the normality of frequency distribution, all variables were subjected to analysis of variance using a one-way ANOVA of the statistical package SPSS 23 version. A multivariate linear regression analysis was performed when appropriate. The differences between means were considered significant for at least *p* < 0.05. The results are presented as mean ± standard deviation.

## 3. Results

All rabbits survived to the 80-day treatments; neither side effect occurred.  Table 1 shows the baseline characteristics in the blood profile divided by groups. No differences were found among groups at the start of the study in glycaemic, lipid, hepatic, and renal profiles (Table 1) and in oxidant-antioxidant levels as shown by ROMs, TBARs, and vitamin A and E levels (Table 2). By using the delta of all parameters, measured as differences between the posttreatment and the baseline levels, significant differences were found among groups. All plant extracts were able to improve blood lipid, glycaemic, and hepatic parameters, with different efficacies among them.

In particular, VB was characterized by the greatest improvement in the lipid profile. In fact, a significant reduction in triglyceride level was found in VB versus CN (*p* < 0.0001) and VB versus RAP (*p* = 0.038) but not with respect to LYC (*p* = 0.055) (Table 3). Similarly, the delta of total cholesterol was the lowest in VB with significant differences in respect to CN and LYC (both *p* < 0.0001) (Table 3). Also, RAP was able to significantly reduce the delta of total cholesterol in respect to CN (*p* < 0.0001) and LYC (*p* = 0.026) (Table 3). All treatments also reduced LDL-cholesterol values in respect to CN (*p* < 0.0001). On the contrary, VB supplementation was the most robust tool to increase HDL cholesterol, with significant differences in respect to all other groups (all *p* < 0.0001) (Table 3). Moreover, VB induced the highest reduction in glycaemic levels in respect to CN, RAP, and LYC (all *p* < 0.0001). Also, LYC showed a significant reduction in glycaemic levels in respect to CN (*p* = 0.007) but not to RAP (*p* = 0.578) (Table 3). The RAP group showed only a partial decrease in respect to CN, which did not achieve any significant value (Table 3).

All plant extracts induced a reduction in the hepatic parameters, sGOT and sGPT, in respect to CN. VB again induced the highest reduction of sGOT (*p* < 0.0001), while no significant differences in reducing sGPT levels among the treatment groups were found (Table 4). No significant differences were found among the treatment groups in respect to CN and to each other in inducing total bilirubin and creatinine level variations (Table 4). In regard to oxidant and antioxidant changes, all plant extracts were able to induce a significant reduction in oxidant levels measured as ROMs and TBARS and an increase in antioxidant vitamins A and E in respect to CN (all *p* < 0.0001). No statistically significant differences were found in ROM levels among the treatment groups, whereas VB showed the highest reduction and RAP the lowest. VB was also responsible for the most important changes in TBARS, with statistically significant differences in respect to RAP (*p* = 0.001) and LYC (*p* = 0.046). No differences were found between RAP and LYC.

In antioxidants, VB was the most effective in increasing both vitamins A and E in respect to LYC (*p* < 0.0001 and *p* = 0.046, resp.), without differences with RAP. However, the RAP group showed higher values in vitamin A changes in respect to LYC (*p* < 0.0001).

**Figure 1:**
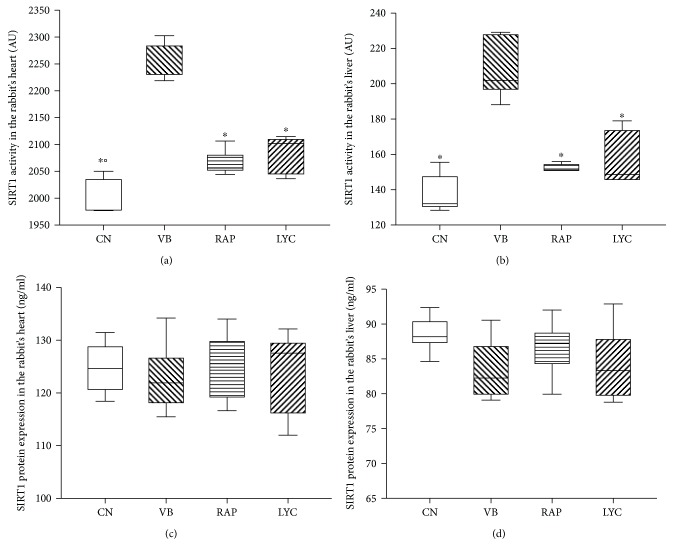
SIRT1 activity in the rabbit's heart (a) and liver (b) and SIRT1 protein expression in the rabbit's heart (c) and liver (d), divided by groups. CN: control; VB: verbascoside; RAP: raphan; LYC: lycopene. ^∗^VB versus other groups (*p* < 0.0001); °LYC versus CN (*p* = 0.013). SIRT1 activity was expressed as arbitrary unit (AU).

Significant differences were also found among groups in SIRT1 activity measured in the rabbit's heart and liver, with VB showing higher levels in respect to CN, RAP, and LYC (all *p* < 0.0001, Figures 1(a) and 1(b)). No significant differences were found between the RAP and CN groups in SIRT1 activity in both the heart and liver, whereas higher values characterized the RAP in respect to the CN group. Finally, in the heart, LYC showed significantly higher SIRT1 activity in respect to CN (*p* = 0.013) but not to RAP (Figure 1(a)), while in the liver, no differences were found (Figure 1(b)). By SIRT1 protein expression evaluation, no differences were found between groups in both the heart (Figure 1(c)) and liver (Figure 1(d)) of the rabbits.

**Figure 2:**
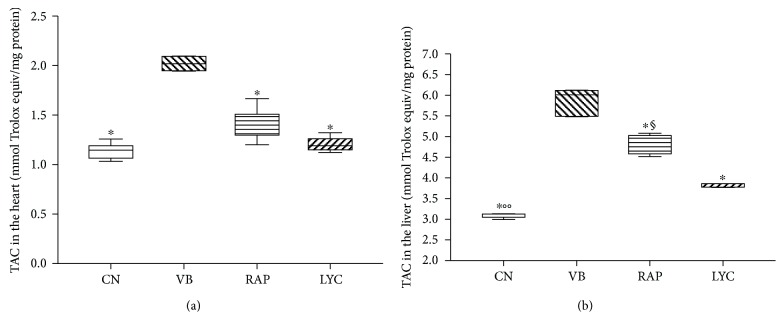
Total antioxidant activity (TAC) by the ABTS assay in the rabbit's heart (a) and liver (b), divided by groups. CN: control; VB: verbascoside; RAP: raphan; LYC: lycopene. ^∗^VB versus other groups (*p* < 0.0001); ^§^RAP versus LYC (*p* < 0.0001); °°LYC versus CN (*p* = 0.005). The ABTS assay was expressed as mmol Trolox equivalent/mg protein.

The total antioxidant capacity (TAC) of all plant extracts was also checked by the ABTS assay. In the heart, VB was the only extract able to significantly increase (*p* < 0.0001) TAC in respect to the CN group, without any differences among RAP, LYC, and CN groups (Figure 2(a)). In the liver, significant differences (*p* < 0.0001) of TAC were found among all groups in respect to CN, with the highest levels in VB (*p* < 0.0001), followed by RAP (*p* < 0.0001) and LYC (*p* = 0.005) (Figure 2(b)).

**Figure 3:**
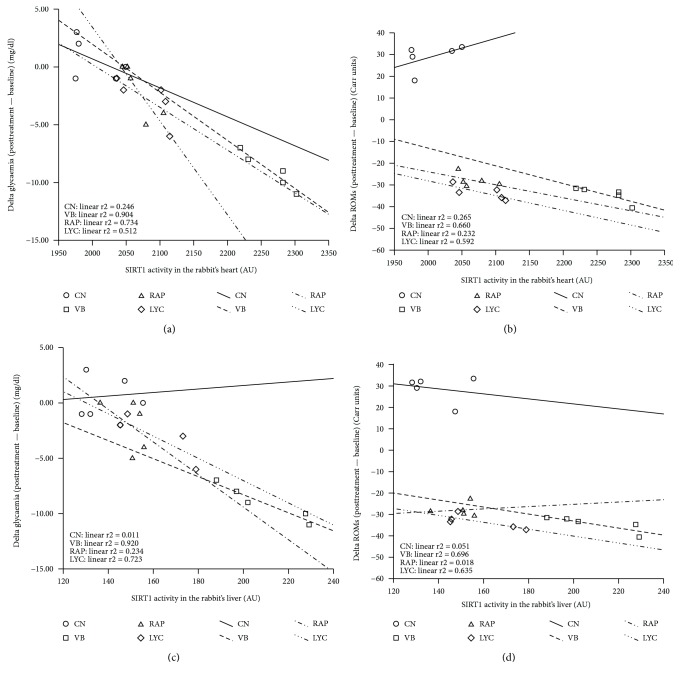
Linear regression by groups between the best predictor delta glycaemia (a) and delta ROMs (b) in the rabbit's heart and between delta glycaemia (c) and delta ROMs (d) in the rabbit's liver. CN: control; VB: verbascoside; RAP: raphan; LYC: lycopene. SIRT1 activity was expressed as arbitrary unit (AU).

By multivariate linear regression analysis, introducing the SIRT1 activity in heart as the dependent variable, the best predictors were represented by the VB group (*p* < 0.0001, *β* = −39.490; 95% CI −55.990, −23.001), the delta glycaemia (*p* < 0.0001, *β* = −18.347; 95% CI −23.276, −13.418; Figure 3(a)), the delta ROMs (*p* = 0.004, *β* = −1.662; 95% CI −2.677, −0.646; Figure 3(b)), and the delta TBARs (*p* = 0.042, *β* = −84.096; 95% CI −164.686, −3.505). Performing the multivariate linear regression analysis, introducing the SIRT1 activity in the liver as the dependent variable, the best predictors were represented by the VB group (*p* < 0.0001, *β* = −29.749; 95% CI −40.941, −18.557), the delta glycaemia (*p* = 0.001, *β* = −3.742; 95% CI −5.714, −1.769; Figure 3(c)), and the delta ROMs (*p* < 0.0001, *β* = −2.23; 95% CI −3.148, −1.257; Figure 3(d)).

## 4. Discussion

Our data demonstrated the efficacy of different plant extracts on lipid and glycaemic profiles and oxidant/antioxidant status (e.g., TBARS, ROMs, TAC, and vitamins A and E). In particular, the beneficial effects of VB seem to be mediated by SIRT1 activity in both the rabbit's heart and liver, revealing for the first time the existence of a direct correlation between the VB beneficial effects and increase in SIRT1 activity.

Multiple aspects of cellular and redox signalling in response to cellular stressors are finely regulated and conserved in both animals and plants [25–27].

In this study, we suggest that the different effects of the plant extracts could be related to their diverse chemical composition. The most important benefits were obtained by a treatment with *Lippia citriodora* containing verbascoside 5 mg/kg feed.

Verbascoside is a polyphenol belonging to the phenolic acid subclass used as a medicinal plant, by virtue of a wide spectrum of biological activity including cardiovascular effects [28, 29].

In our study, the evidence that verbascoside was able not only to reduce all parameters involved in cardiovascular risk composition, such as glycaemia, total cholesterol, and LDL cholesterol, but also to induce an increase in HDL cholesterol, a well-identified protective cardiovascular factor, confirmed these effects.

Moreover, the VB effects on vitamin A and E levels reinforce the antioxidant capability of this plant extract. Previously, Rossi et al. showed that plant extracts of Verbenaceae might improve vitamin E status in vivo by protecting *α*-tocopherol from oxidative decay [30]. Similarly, Iglesias et al. [31] reported that the procyanidins could repair oxidized *α*-tocopherol in the medium-long term in fish muscle. Palazzo et al. [32] demonstrated that the increased vitamin A and E levels can be attributed to the ability of VB to strengthen and save the endogenous antioxidant system, controlling oxidative metabolism by reducing the production of reactive radical species and increasing the antioxidant activity of enzymes [33–35]. A dual mechanism of action, proposed by Palazzo et al. [32], includes the direct action of capturing free radicals during the propagation of a chain reaction and the inhibition of prooxidant enzymes as the initiation of the oxidation phase [36]. Vitamin E, in its function as a chain-breaking antioxidant, rapidly transfers its phenolic H-atom to a lipid peroxyl radical, converting it into a lipid hydroperoxide and a vitamin E radical [37–39].

Indeed, a relationship between SIRT1 activity and vitamin E levels was demonstrated in the hippocampus and cerebral cortex of rodents, in which vitamin E prevented a decrease in SIRT1 expression caused by a high-fat diet [40]. Also, Zillikens et al. [41] found an interaction between vitamin E and SIRT1, suggesting that this interface could be due to the antioxidant function of vitamin E and its role as a regulator of enzymes and gene activity [42]. In our knowledge, this is the first study that demonstrated increased levels of vitamins A and E associated with increased SIRT1 activity.

According to Funes et al. [43], the VB biological activity could also be related to its ability to modulate membrane-dependent cellular processes, such as cell signalling [44] and mitochondrial function [45, 46], although the molecular basis of these effects still remains unclear [43].

In addition, Zhang et al. [46] demonstrated that in cells treated with H_2_O_2_, a well-known cellular oxidant, the administration of VB induced a reduction of DNA oxidative damage [46].

Interestingly, VB and its derivatives are the only well-established examples of successful action, due to the concomitant inhibition of expression and DNA binding of the two main proinflammatory transcription factors activator protein-1 and nuclear factor *κ*B [47]. Indeed, the activities of the main antioxidant enzymes catalase, glutathione peroxidase, and glutathione reductase showed to be significantly enhanced by VB [48]. Quirantes-Piné et al. [49] showed that after VB administration, the antioxidant enzyme activities significantly augmented while the myeloperoxidase activity diminished, suggesting VB protective antioxidant effects on blood cells and its potential anti-inflammatory and antiatherogenic activities in rats. Then, the authors suggested that these VB actions influence redox enzymes at the posttranscriptional level [49]. Furthermore, some data have confirmed that the reduced NF-*κ*B activation represents the mechanism responsible for the VB protective effects [48, 50].

The NF-*κ*B system regulates apoptosis, proliferation, and energy metabolism and then survival during cellular stress [51–53]. SIRT1 is able to regulate NF-*κ*B signalling, thereby contributing to the maintenance of cellular homeostasis [54, 55].

In this study, the treatment with VB induced a significant increase in SIRT1 activity in the rabbit's heart, suggesting a possible role of this key redox state regulator in stimulating changes of the oxidant and antioxidant levels. This finding was confirmed by the rise of SIRT1 activity also in the rabbit's liver. We hypothesised that VB is responsible for SIRT1 activation through a hormetic mechanism. The consequent activation of SIRT1 was in turn able to induce the total antioxidant capacity, including enzymes such as catalase and SOD, well-known Sirt1 targets.

Previously, several studies have demonstrated the VB capability to induce an increase in catalase and SOD activity. Recently, Dell'Aquila et al. [56] demonstrated that catalase activity significantly increased in VB-exposed oocytes. The same authors suggested that this increase could be due to the potential formation of H_2_O_2_ during the oxidation of phenolics, concluding that the stimulating effect of VB on CAT activity may also be related to the observed increase in intracellular ROS levels [56]. Also, Sheng et al. [57] revealed that VB significantly decreased apoptotic death in PC12 cells, whereas it increased extracellular hydrogen peroxide levels.

These findings support the hypothesis that a hormetic effect could be responsible for SIRT1 activation, without changes in its expression, and consequent increased antioxidant response.

Finally, the possible explanation of the different behaviours of the other extracts, *Raphanus* and lycopene, on blood profiles could be related to their dissimilar chemical composition. In fact, the horseradish contains polyphenols derived from flavonoids and lignans, whose titration, however, has not been well defined. Lycopene, on the other hand, is a carotenoid. According to our study that showed also an increase in SIRT1 activity in rabbits treated with lycopene with effects on glycaemic and lipid profiles, Luvizotto et al. [58] showed that lycopene supplementation upregulated mRNA expression of SIRT1 in rats.

## 5. Conclusions

This is the first study that demonstrated a relationship between VB administration and SIrt1 activity in the rabbit's heart and liver tissue, suggesting that SIRT1 could mediate the antioxidant effects of this extract. Moreover, these data suggest that all the plant extracts VB, RAP, and LYC in the concentration used are not toxic and able to improve glycaemic and lipid profiles, through induction of antioxidant effects that, in particular for the VB, could be mediated by the SIRT1 activity. Surely, further studies are needed to confirm the possible underlining mechanisms, to investigate the involvement of other sirtuins (such as Sirt3) and to settle the role of hormesis in the antioxidant response induced by these extracts.

## Figures and Tables

**Table 1:** Baseline lipid, glycaemic, hepatic, and renal profiles divided by groups.

	CN	VB	RAP	LYC	*p*
Body weight (gr)	1448 ± 110.32	1532 ± 58.05	1497 ± 51.22	1486.5 ± 64.55	0.302
Triglycerides (mmol/l)	99.08 ± 3.85	101.88 ± 4.77	100.63 ± 6.42	100.8 ± 5.69	0.885
Total cholesterol (mmol/l)	64.38 ± 4.01	64.44 ± 4.82	64.37 ± 4.35	65.12 ± 2.90	0.972
LDL cholesterol (mmol/l)	30.98 ± 2.26	30.5 ± 1.65	30.46 ± 2.71	30.67 ± 1.96	0.978
HDL cholesterol (mmol/l)	25.38 ± 2.32	25.92 ± 1.20	25.6 ± 1.46	25.48 ± 1.89	0.961
Glycaemia (mmol/l)	158.6 ± 6.11	158.6 ± 7.40	158 ± 6.99	156.7 ± 6.04	0.933
sGOT (U/l)	18.12 ± 1.84	18.58 ± 0.88	19.58 ± 2.46	19.54 ± 1.99	0.488
sGPT (U/l)	25.4 ± 3.71	25.8 ± 2.89	25.44 ± 3.09	25.43 ± 2.56	0.996
Total bilirubin (mmol/l)	1.03 ± 0.15	1.01 ± 0.08	0.96 ± 0.13	0.962 ± 0.13	0.659
Creatinin (mmol/l)	1016 ± 0.16	1.10 ± 0.18	1.15 ± 0.16	1.08 ± 0.28	0.722

LDL: low-density lipoprotein; HDL: high-density lipoprotein; sGOT: serum glutamic oxaloacetic transaminase; sGPT: serum glutamic pyruvic transaminase.

**Table 2:** Baseline oxidant-antioxidant levels in blood of the rabbits divided by groups.

	CN	VB	RAP	LYC	*p*
ROMs (Carr units)	153.62 ± 15.64	155.21 ± 12.96	151.01 ± 17.34	155.06 ± 15.93	0.940
TBARS (*μ*mol/l)	1.31 ± 0.14	1.34 ± 0.27	1.32 ± 0.22	1.32 ± 0.22	0.998
Vitamin A (*μ*mol/l)	8.93 ± 1.04	8.25 ± 1.05	8.34 ± 1.30	8.77 ± 1.14	0.676
Vitamin E *μ*mol/l)	0.66 ± 0.09	0.64 ± 0.11	0.65 ± 0.12	0.64 ± 0.09	0.991

ROMs: reactive oxygen metabolites; TBARS: thiobarbituric acid reactive substances.

**Table 3:** Blood parameter variations on the basis of different treatments.

	CN	VB	RAP	LYC	*p*
ΔTriglycerides (mmol/l)	0.64 ± 144^∗§°^	−10.74 ± 1.47	−8.01 ± 1.89^∗∗^	−8.15 ± 1.64	<00001
ΔTotal cholesterol (mmol/l)	1.28 ± 0.4^∗§°^	−6.84 ± 0.77	−5.51 ± 0.91	−4.21 ± 1.07^∗∗∗§§^	<0.0001
ΔLDL cholesterol (mmol/l)	1.92 ± 1.62^∗§°^	−5.06 ± 0.81	−3.46 ± 0.98	−4.2 ± 0.91	<0.0001
ΔHDL cholesterol (mmol/l)	0.12 ± 0.19^∗§°°^	2.68 ± 0.25	1.4 ± 0.47^∗∗∗∗^	0.91 ± 1.80^∗∗∗§§§^	<0.0001
ΔGlycaemia (mmol/l)	−0.2 ± 2.75^∗°°°^	−9.0 ± 1.58	−1.5 ± 1.96^∗∗∗∗^	−3.0 ± 1.83^∗∗∗^	<0.0001
ΔsGOT (U/l)	0.38 ± 0.78^∗§°°^	−2.52 ± 0.98	−2.15 ± 0.56^∗∗∗∗∗^	−1.66 ± 0.97^∗∗∗^	<0.0001
ΔsGPT (U/l)	1.58 ± 1.13^∗§°^	−3.66 ± 0.88	−1.9 ± 0.89	−0.89 ± 0.80	<0.0001
ΔTotal bilirubin (mmol/l)	−0.0180 ± 0.15	−0.16 ± 0.15	−0.05 ± 0.37	−0.52 ± 0.04	0.075
ΔCreatinin (mmol/l)	0016 ± 0.07	−0.16 ± 0.14	0.15 ± 0.13	−0.15 ± 0.12	0.082

Δ: differences measured between posttreatment and baseline; LDL: low-density lipoprotein; HDL: high-density lipoprotein; sGOT: serum glutamic oxaloacetic transaminase; sGPT: serum glutamic pyruvic transaminase. ^∗^VB versus CN (*p* < 0.0001); ^∗∗^VB versus RAP (*p* = 0.038); ^∗∗∗^VB versus LYC (*p* < 0.0001); ^∗∗∗∗^VB versus RAP (*p* < 0.0001); ^∗∗∗∗∗^VB versus RAP (*p* = 0.010). ^§^RAP versus CN (*p* < 0.0001); ^§§^RAP versus LYC (*p* = 0.026); ^§§§^RAP versus LYC (*p* = 0.011). °LYC versus CN (*p* < 0.0001); °°LYC versus CN (*p* = 0.001); °°°LYC versus CN (*p* = 0.007).

**Table 4:** Oxidant-antioxidant level changes in blood of the rabbits divided by groups.

	CN	VB	RAP	LYC	*p*
ΔROMs (Carr units)	28.84 ± 6.24^∗§°^	−34.49 ± 3.62	−29.81 ± 4.01	−33.99 ± 3.26	<0.0001
ΔTBARS (*μ*mol/l)	0.54 ± 0.19^∗§°^	−0.62 ± 0.06	−0.41 ± 0.05^∗∗^	−0.48 ± 0.05^∗∗∗^	<0.0001
ΔVitamin A (*μ*mol/l)	−0.21 ± 0.09^∗§°^	8.49 ± 1.02	8.19 ± 1.31^∗∗∗∗^	5.30 ± 1.3^∗∗∗∗∗§§^	<0.0001
ΔVitamin E (*μ*mol/l)	−0.033 ± 0.014^∗§°^	0.64 ± 0.08	0.59 ± 0.10	0.48 ± 0.12^∗∗∗^	<0.0001

Δ: differences measured between posttreatment and baseline; ROMs: reactive oxygen metabolites; TBARS: thiobarbituric acid reactive substances. ^∗^VB versus CN (*p* < 0.0001); ^∗∗^VB versus RAP (*p* = 0.001); ^∗∗∗^VB versus LYC (*p* = 0.046); ^∗∗∗∗^VB versus RAP (*p* < 0.0001); ^∗∗∗∗∗^VB versus LYC (*p* < 0.0001). ^§^RAP versus CN (*p* < 0.0001); ^§§^RAP versus LYC (*p* < 0.0001). °LYC versus CN (*p* < 0.0001).

## Data Availability

The data used to support the findings of this study are available from the corresponding author upon request.
